# End of the first decade of EHJ CVP

**DOI:** 10.1093/ehjcvp/pvaf081

**Published:** 2025-12-16

**Authors:** Stefan Agewall

**Affiliations:** Institute of Clinical Sciences, Karolinska Institute of Danderyd, Stockholm, Sweden

**Dear Reader,**

**Figure:**
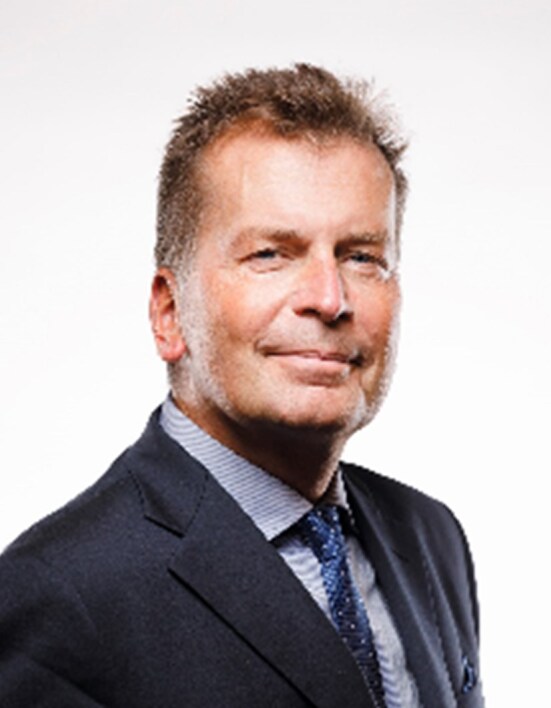


Looking back, it feels a bit like herding cats—but we actually did it.

When I joined the ESC Working Group on Cardiovascular Pharmacotherapy board back in 2010, none of the ESC working groups had their own journal. Many were affiliated with other publications, but we wanted to be pioneers—to create something that was truly ours. The idea took shape in 2013, and with a small but determined crew, we began sketching out what would become *European Heart Journal—Cardiovascular Pharmacotherapy*.

We worked closely with the ESC and Oxford University Press (OUP), and I still remember the countless meetings, long evenings, and endless email chains. With the steady support of Michael Alexander from ESC and the tireless OUP team, our dream slowly materialized into reality. When I was elected Editor-in-Chief in 2014, I felt equal parts honored and terrified. My first companions on this editorial adventure were Basil Lewis, Dan Atar (who later stepped down when he became EiC of *Cardiology* in 2019), and Keld Kjeldsen. Over time, we built a diverse editorial board, spanning continents, subspecialties, and genders—truly a global family.

After a flurry of paperwork, we decided to print a promotional issue. I reached out (perhaps too eagerly!) to the biggest names in cardiology, hoping they’d lend credibility to our fledgling journal—and to my great relief, many of them said yes. We received contributions from TF Lüscher, E Braunwald, GYH Lip, GMC Rosano/C Ceconi, and F Zannad. Not a bad lineup for a newborn journal without an impact factor or indexing.

Our first official issue launched in 2015 as part of the ESC journal family, proudly published by OUP. The early challenge was obvious—how do you convince top researchers to submit to a journal that is not indexed nor has an impact factor? I must have written to every familiar name in cardiology (and perhaps a few tried to avoid eye contact with me at conferences to not get the question if they had some paper to submit). Yet, slowly but surely, submissions came in, and we began to grow. Having ‘European Heart Journal’ in our title certainly didn’t hurt!

A new journal typically needs 2–3 years of consistent publication before even being considered for indexing in major databases such as SCI Expanded or Medline. We made it in just 2 years. Our first Impact Factor arrived in 2019 (for 2018 data), which is remarkably fast for a niche cardiovascular pharmacotherapy journal. Today, our Impact Factor remains stable at 6.1—a testament to the quality of work our authors deliver and our reviewers help uphold.

And best of all? I’m no longer that pariah begging for manuscripts in conference corridors. We now reject over 90% of submissions, and instead, authors ask *us* if they may submit their next paper. How’s that for a role reversal?

In 2024, we announced that the journal would transition to full open access from January 2025, with all manuscripts submitted after 3 September 2024 published under an OA license. I braced for a potential decline in submissions—but to my pleasant surprise, the opposite happened. Submissions have increased, reaffirming the enthusiasm and relevance of our field.

The growth of *EHJ CVP* reflects the expanding role of pharmacotherapy in cardiology. Much of the cardiovascular progress over recent decades—from novel antidiabetic agents to lipid-lowering and anticoagulant drugs—stems directly from pharmacological innovation. Our journal has become the dedicated home for that research, ensuring these advances are recognized and shared within the ESC community and beyond.

After 11 years as Editor-in-Chief, it’s time for me to pass the baton. It has been a privilege to steer this journal from an idea into a respected member of the ESC family. I wish my successor—and the journal—continued success. And who knows—perhaps when we celebrate the 20-year anniversary, *EHJ CVP* will boast an impact factor above 10. I certainly wouldn’t bet against it.

## In this issue

This issue features several *PharmaPulse* summaries from recent large-scale clinical studies, alongside a comprehensive ESC Working Group review.

## Rivaroxaban vs. apixaban in NVAF with PAD

Peripheral artery disease (PAD) increases the risk of stroke and cardiovascular death in patients with nonvalvular atrial fibrillation (NVAF).^1,2^ In a UK cohort study (2013–2021; *n* = 16 160), Renoux *et al*. compared rivaroxaban and apixaban in NVAF patients with PAD. Propensity weighting balanced baseline factors. Rates of stroke/TIA/systemic embolism were similar, but rivaroxaban carried a higher major-bleeding risk. Both agents were equally effective, yet apixaban offered superior safety.

## Ranolazine in chronic coronary syndromes

Ranolazine, a late Na^+^-current inhibitor approved for chronic coronary syndromes (CCS), may also have atrial-selective antiarrhythmic properties.^3^ Fumagalli *et al*. analyzed 171 015 CCS patients from Italian NHS data (2011–2020); 22 207 received ranolazine. After propensity-score matching (Ran = 6 384; No-Ran = 25 536), ranolazine use was linked to a 26% reduction in all-cause mortality (HR 0.74; *P* < 0.001), fewer atrial and ventricular arrhythmias, and lower AF incidence. The findings support ranolazine’s safety and call for randomized trials to confirm its antiarrhythmic benefits.

## Postoperative atrial fibrillation and stroke

Postoperative atrial fibrillation (POAF) has often been dismissed as transient and benign, yet accumulating data indicate otherwise.^4^ De Catarine *et al*. conducted a systematic review and meta-analysis of 40 studies (19 in meta-analysis), revealing a threefold increased risk of stroke following noncardiac surgery among POAF patients. Variability across surgical types and patient profiles highlights the need for better ECG monitoring and prospective trials on anticoagulation.

## Dyslipidemia in type 2 diabetes Mellitus

Drexel *et al*., together with the ESC WG on Cardiovascular Pharmacotherapy, present a review on managing dyslipidemia in T2DM patients.^5-9^ Diabetic dyslipidemia—characterized by high triglycerides, low HDL-C, and small dense LDL particles—remains a key driver of atherosclerotic cardiovascular disease (ASCVD). Statins remain foundational, with ezetimibe and PCSK9 inhibitors providing additive benefits. Despite modest glycemic effects, their cardiovascular protection is undeniable. Guidelines now emphasize LDL-C and non-HDL-C targets with intensity guided by total risk stratification.

## Anti-inflammatory pharmacotherapy in patients with cardiovascular disease

An invited review by Dr Capodanno *et al*. highlights inflammation as a central mechanism in cardiovascular disease (CVD), influencing atherosclerosis, plaque rupture, and myocardial injury.^10,11^ Colchicine has shown benefit in stable CAD, though recent trials raise questions about its post-MI role.^12^ Monoclonal antibodies targeting IL-1β, IL-6, and IL-6R show promise but remain investigational. Emerging therapies aim for precision with minimal immunosuppression, suggesting future strategies will integrate inflammation control with lipid and thrombotic risk management
